# Mapping intralobar fiber connections in the human occipital lobe by tracer electrophoresis

**DOI:** 10.1007/s00429-025-03031-2

**Published:** 2026-01-06

**Authors:** Lars Freudenmacher, Horst-Werner Korf, Svenja Caspers

**Affiliations:** 1 Institute for Anatomy I, Medical Faculty & University Hospital Düsseldorf, Heinrich Heine University, Düsseldorf, Germany; 2 Institute of Neuroscience and Medicine (INM-1), Research Centre Jülich, Jülich, Germany

**Keywords:** Human visual cortex, Tract tracing, Lipophilic tracer, Tracer electrophoresis

## Abstract

The cellular connectivity of the human visual cortex remains largely uncharted, with most insights derived from animal models. A custom-designed tracer electrophoresis setup for polar lipophilic tracers enabled the reconstruction of fiber tracts in fixed postmortem human brains, visualizing their trajectories across previously unmapped distances, from cellular origins to axonal terminations. Applied to the occipital lobe, four pathways of the calcarine cortex were demonstrated: the *stratum calcarinum*, *stratum proprium cunei*, *fasciculus transversus cunei*, and the *fasciculus transversus gyri lingualis*. Specifically, axonal projections from granular and supragranular layers of the calcarine cortex were traced to the granular and supragranular layers of the cuneus, inferior, middle, and superior occipital, lingual, and fusiform gyri. Additionally, infra- and supragranular feedback projections from the prestriate cortex to the supragranular layer of the calcarine cortex were identified. These results extend previous descriptions by offering the first cellular-level evidence for intrahemispheric pathways in the human occipital lobe.

**Supplementary Information:**

The online version contains supplementary material available at 10.1007/s00429-025-03031-2.

## Introduction

Understanding the structural connectivity of the human brain remains a fundamental challenge in neuroscience. The visual cortex, one of the most extensively studied systems, serves as a crucial model for exploring broader network architecture. However, much of our current knowledge is derived from non-human primate studies, such as the comprehensive work of Felleman and Van Essen (1991) and its further refinement in network-based analyses by Hilgetag et al. (2016). Achieving a comparable level of detail in humans has proven elusive due to significant methodological limitations.

The primary challenge in mapping cortical connectivity at cellular resolution in humans lies in the inapplicability of invasive techniques, such as autoradiographic or viral tract tracing, which require living tissue and are therefore limited to animal studies (Rushmore et al. 2020). While non-invasive methods, such as diffusion-weighted imaging (DWI), provide valuable large-scale insights, they lack the spatial resolution needed to reliably distinguish small, intersecting fiber tracts. Postmortem dissection studies, though informative for gross anatomical organization, fail to resolve fine-scale cortical connectivity and laminar specificity. Similarly, histological approaches such as 3D-polarized light imaging cannot trace intercortical connections with the necessary cellular precision (Caspers et al. 2015). As a result, fundamental aspects of fiber origins, trajectories, and terminations remain insufficiently characterized in humans, particularly in regions with complex fiber architectures.

Traditional human postmortem tract-tracing techniques, which rely on passive diffusion of lipophilic tracers within neuronal membranes, are constrained by slow diffusion rates and limited tracing distances. Up to three centimeters have been achieved in peripheral nerves and spinal cord within 15 weeks (Lukas et al. 1998), yet in the human cortex distances beyond one centimeter have not been reported (Tardif and Clarke 2001; Mufson et al. 1990; Thal et al. 2008; Supplementary Table 1). Accordingly, human postmortem tracing has remained focused on local connections, emphasizing the need for methods capable of demonstrating longer-range pathways within shorter times.

Electrophoresis of polar tracers has improved the tracing distance in the peripheral nervous system and enabled the visualization of axons across five to six centimeters within 48 h (Madison et al. 2014; Isaacson and Hedwig 2017; Swift et al. 2005). However, applying a directional electric field to the human brain presents additional challenges due to the multidirectional orientation of fiber tracts. To address this, we developed Rotating Field Tracer Electrophoresis (RFTE), which combines an applied electric potential with continuous reorientation of the brain sample to facilitate tracer movement along different fiber orientations.

 The occipital lobe, in particular the calcarine cortex, offers a suitable model to evaluate RFTE. Its intrahemispheric association pathways have been extensively documented in classical anatomical work (Wernicke 1881; Sachs 1892; translated by Forkel et al. 2015; Vialet 1893a, b; Déjerine and Déjerine-Klumpke 1895) and revisited in modern imaging and dissection studies (Takemura et al. 2019; Bugain et al. 2021; Latini et al. 2015; Vergani et al. 2014). Five major pathways are described: (i) the *stratum proprium cunei*, a short sagittal tract linking the cuneus with the superior occipital gyrus; (ii) the *fasciculus transversus cunei*, a transverse tract above the lateral ventricle linking the cuneus with lateral and inferior occipital regions; (iii) the *stratum calcarinum*, a U-shaped bundle interconnecting the cuneus and lingual gyrus; (iv) the *fasciculus transversus gyri lingualis*, an oblique tract below the lateral ventricle linking the lingual gyrus with ventral occipital areas; and (v) the *vertical occipital fascicle*, a lateral vertical bundle linking dorsal and ventral occipital cortices. However, due to technical limitations, the transverse fascicles of the cuneus and lingual gyrus remain incompletely resolved (Bugain et al. 2021; Vergani et al. 2014), underscoring the need to define occipital connectivity at the cellular level.

Because most fibers of these association pathways run within the plane of a coronal section, the occipital lobe provides a practical benchmark. RFTE can be regarded as reliable if (i) tracer applications into different parts of the calcarine cortex yield gradual shifts in tract labeling, (ii) the trajectory of a tract can be followed to its axonal termination in the cortex, (iii) complementary applications in connected regions label the same tracts in reverse and demonstrate their cellular origin, and (iv) the resulting connections corresponds to the known laminar organization from primate data (Felleman and Van Essen 1991; Hilgetag et al. 2016). Unrelated fibers should not be labeled and serve as internal negative controls. Under the null hypothesis, labeling would disregard these anatomical constraints and appear diffuse or indiscriminate. By comparing human and animal data, the occipital lobe thus provides both a framework to validate RFTE and an opportunity to address open questions in human intercortical connectivity.

## Materials and methods

### Human material

Postmortem human brains (*n* = 8) and peripheral nerve samples (*n* = 3) were obtained from nine adult body donors (both sexes) of the body donor program of the Anatomy Department of the University of Düsseldorf, according to respective legal and ethical guidelines and approval of the local Ethics Committee (#2022-2264). The donors had no clinical record of neurological or psychiatric illnesses. The brains and nerve samples were collected within 24 h after determination of death. The brains are designated B1–B8 in the following.

### Protocol development

To establish a stable protocol for RFTE in human brain tissue, several approaches were tested to optimize the distribution, preservation, and quality of *FAST*-DiI tracer labeling. Earlier studies reported that cryostat sectioning, drying, heating, calcium exposure, and glycerol-containing mounting media can induce leakage and loss of specific labeling, complicating systematic evaluation of lipophilic tracers (Lukas et al. 1998; Friedman et al. 1991; Hofmann and Bleckmann 1999; Murphy and Fox 2007; Makarenko et al. 2001; Holmqvist et al. 1992; Supplementary Table 1). In this study, critical parameters included fixation method, as well as the duration and voltage of electrophoresis, which influenced tracer uptake and diffusion. Sectioning method, storage, and mounting medium determined the long-term stability of labeling, the feasibility of obtaining large histological sections without tissue loss, and therefore the ability to reconstruct individual pathways across consecutive histological sections. A reproducible standard protocol was established, balancing labeling quality with feasibility for large-scale reconstruction. The protocol included the following steps: short fixation with a 4% formaldehyde (FA) solution, decalcification, tracer application, tracer electrophoresis, and cryostat sectioning. To prevent signal loss, cryostat sections were stored at – 80 °C. A custom mounting medium containing α-thioglycerol proved essential for preserving fluorescence and enabling systematic analysis. Mounted sections were subsequently stored at 4 °C until evaluation. Alternative protocols that were tested during protocol development are summarized in Table 1.

### Tissue fixation

For each brain (B1–B8), two to three coronal sections (S1-S3; occipital to frontal; 1–3 cm thick) were prepared from the left and right hemisphere (L/R). Sections with visible white matter damage from cutting or processing were excluded. In the standard protocol, coronal brain sections and peripheral nerve tissue were prepared prior to short fixation (2 h–6 d) to avoid over-fixation, which can impair tracer diffusion (Godement et al. 1987; Sparks et al. 2000; Chen et al. 2006; Supplementary Table 1). Fixation was performed using freshly prepared 4% FA solution made of paraformaldehyde (PFA) with ethylenediaminetetraacetic acid (EDTA) for decalcification (4% [wt/vol] PFA, 0.1% [wt/vol] EDTA in 0.1 M phosphate-buffer [PB] at pH 7.4). Alternative fixations (AF) are summarized in Table 1. These included fixation of whole brains before sectioning with prolonged fixation times (AF1, 1 month to 2 years), or fixation with 3% periodate-lysine-paraformaldehyde (AF2, PLP; 3% [wt/vol] PFA, 0.8% [wt/vol] sodium periodate, 0.1 M D-L-lysine in 0.1 M phosphate-buffered saline [PBS] at pH 7.4), which has been proposed to facilitate DiI diffusion (Hildebrand et al. 2020; Supplementary Table 1).

### Tracer application

The cationic lipophilic membrane stain *FAST*-DiI (Invitrogen™) was dissolved in dimethylformamide (DMF) to create a 10% (wt/vol) stock solution. For tracer application, 1 µl of a 1% working solution was pressure-injected using a Hamilton syringe (needle gauge 26s). Small deposits were used to ensure localized labeling and to avoid unspecific spread into surrounding tissue or fibers of passage. Non-localized tracer applications that did not meet this criterion were excluded from subsequent analysis. Applications were made at different sites of the calcarine cortex (CalCx), including the upper cuneal bank (CalCx–Cu), the floor of the calcarine sulcus (CalCx–F, when present as a distinct deep bank), and the lower lingual bank (CalCx–LG). In addition to these primary applications, complementary injections were placed in the white matter of the superior (SOG), middle (MOG), and inferior (IOG) occipital gyri. All samples were stored in PB (0.1% EDTA, 0.01% NaN₃) at 4 °C until further processing.

### Control experiments

To validate the specificity of RFTE, several control experiments were performed:

1. **Tracer diffusion without electrophoresis**: 1 µl of 1% *FAST*-DiI was injected into the calcarine cortex and incubated in PB (0.1% EDTA, 0.01% NaN₃) for three weeks to six months at room temperature.
2. **Assessment of potential tracer spill**: Occipital lobe tissue samples were incubated with 1 µl of 1% *FAST*-DiI in 100 ml of PB (0.1% EDTA, 0.01% NaN₃) for three weeks at room temperature.
3. **Negative control**: Unstained occipital lobe tissue was processed in parallel to check for autofluorescent background signal.
4. **Study replication** (Swift et al. 2005): 1 µl of 1% *FAST*-DiI was applied to the cut proximal end of peripheral (tibial and common fibular) nerves, followed by tracer electrophoresis.

### RFTE chamber

**Fig. 1:**
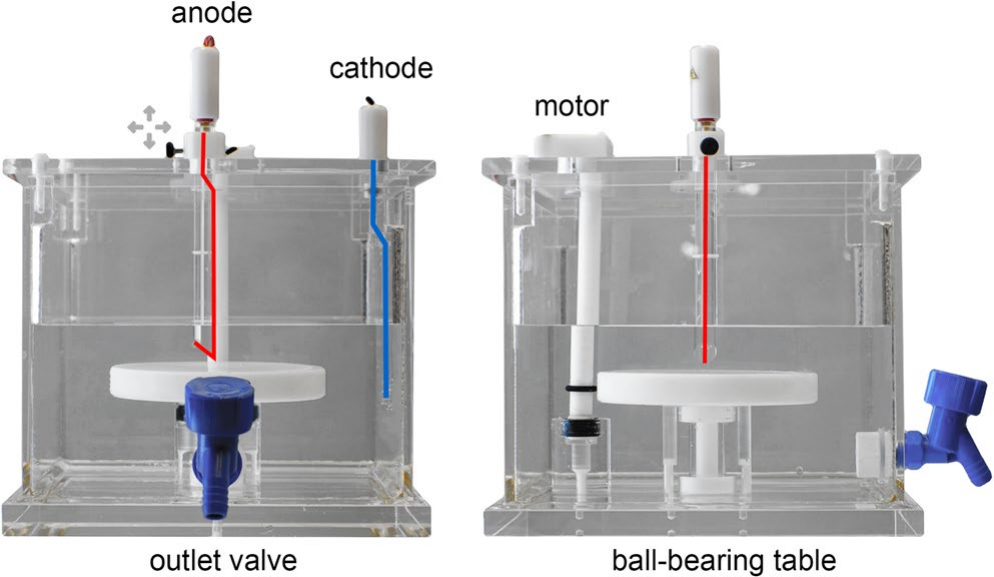
Rotating field tracer electrophoresis (RFTE) chamber. The electrodes are oriented vertically, the anode (red) is located centrally and can be varied in position, the cathode (blue) is mounted on the side of the chamber. A rotating platform, mounted on plastic ball-bearing, is driven with a stepping motor via a friction fit

To enable fast distribution of polar fluorescent tracers along different fiber orientations, we developed a custom tracer electrophoresis chamber (Fig. 1). The chamber is square, made from plexiglass and has a volume of approximately 10 l. A rotating platform, mounted on plastic ball-bearing, is attached in the center of the chamber. The platform is driven by a stepper motor via a pivot-bearing axle with a friction fit. The stepper motor in turn is controlled by a microcontroller (Arduino) and changes its direction after several rotations. The platform has a silicone surface, allowing the fixation of a tissue sample with non-conductive needles. The electrodes are vertically oriented and made of platinum wire. The anode is attached to the lid of the chamber and is suspended centrally above to the platform. For fine adjustment, the anode can be varied horizontally and vertically using set screws. The cathode is attached to the side of the chamber. The chamber can be emptied by an outlet valve. For safety reasons, the contacts to the power supply get disconnected when the lid is removed. Furthermore, the setup is secured by a water detector with a power cut-off.

### RFTE protocol

Following tracer application, the tissue was placed on the rotating platform inside the electrophoresis chamber, with the tracer application site aligned centrally to the anode. The chamber then was filled with TRIS-borate-EDTA buffer (TBE). A large buffer volume (6–8 l) was used to prevent buffer depletion and improve heat dissipation. To avoid heat-induced tissue damage, the electrophoresis chamber was placed in a refrigerator at 4 °C. In the standard protocol, electrophoresis was performed at 80 V for 106–168 h using a Biometra Standard Power Pack P25. During electrophoresis, the tissue temperature remained below 30 °C. After electrophoresis, the samples were stored in PB (0.1% EDTA, 0.01% NaN₃) at 4 °C until further processing. Alternative electrophoresis parameters (EP) are summarized in Table 1.

### Optional tissue embedding

**Fig. 2:**
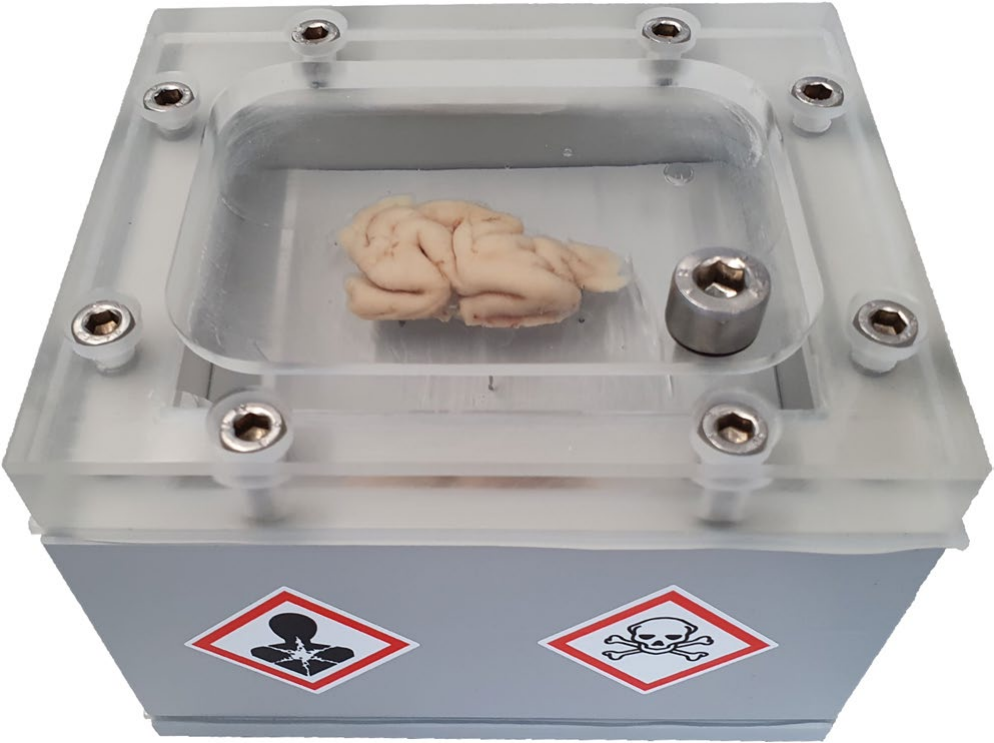
Acrylamide embedding chamber. The chamber has a screwable lid and bottom, sealed by silicone sheets. The lid has an overflow tray, allowing the chamber to be filled without air bubbles via a filler neck. The filler neck is closed with a screw and a rubber seal. The volume of the chamber can be varied via plastic inserts. Blunt thin metal pins in the uppermost insert provide a separation to the bottom of the chamber. This ensures that the tissue is suspended in the acrylamide-solution and that it is completely embedded inside the hydrogel after polymerization

Embedding was not part of the standard protocol. For vibrating-blade microtome sectioning, two alternative embedding (AE) protocols were tested: Agarose hydrogel embedding (AE1): For embedding, samples were ice-cooled on a metal plate and the hot agarose solution (4% [wt/vol] agarose, 0.1% EDTA in PB) was cooled down just before the consolidation point. Polyacrylamide hydrogel embedding (AE2; Hayaran and Bijlani 1992; Supplementary Table 1): To prevent oxygen inhibition of polymerization, the tissue was placed in a custom airtight embedding chamber (Fig. 2) and infiltrated for 24 h with a 10% acrylamide monomer solution prepared from a 40% acrylamide/bisacrylamide (37.5:1) stock. Polymerization was initiated by adding 150 µl ammonium persulfate (APS; 10% [wt/vol]) and 30 µl tetramethylethylenediamine (TEMED) to 100 ml of solution. The chamber was kept at 4 °C for 24–48 h to allow slow and uniform polymerization, followed by incubation at 37 °C for 2 h to remove residual acrylamide. After polymerization, the tissue was fully enveloped in hydrogel and acquired a stable, rubber-like consistency.

### Sectioning

Following electrophoresis, the tissue was cut into smaller blocks before sectioning. In the standard protocol, cryostat sectioning was performed, as this remains the most viable option for large samples with currently available commercial equipment. Tissue blocks (40 × 55 mm) were cryoprotected with 30% sucrose in PB (0.1% EDTA, 0.01% NaN₃) for at least three days, then frozen on a metal plate at – 80 °C. Sections (20–30 μm) were prepared using a Leica CM3050 S cryostat, mounted on adhesion slides, and rapidly refrozen to prevent defrosting or drying. This approach avoided heat-drying of the sections onto the slides, resulting in weaker adhesion but ensuring stable *FAST*-DiI fluorescence signals (Table 1). As an alternative sectioning protocol (AS; Table 1), tissue blocks (30 × 30 mm) were sectioned (60–100 μm) using a Leica VT1200 vibrating-blade microtome. This required prior embedding of the tissue. The block size corresponded to the maximum cutting window of the microtome and was therefore technically demanding due to vibrations, uneven section thickness, and reduced mechanical stability of the tissue.

### Storage conditions

In the standard protocol, cryostat sections were continuously kept frozen at – 80 °C to prevent defrosting or drying. Under this condition, *FAST*-DiI fluorescence remained stable for several months. Alternative storage conditions (ST) are summarized in Table 1.

### Mounting medium

Both the established standard protocol and a set of tested mounting media (MM) were evaluated on consecutive sections from the same preparation. In the standard protocol, sections were mounted in Mowiol^®^ 4-88 prepared according to the manufacturer’s instructions, with glycerol replaced by α-thioglycerol (DiI-compatible chemical; Hildebrand et al. 2020). The medium was supplemented with the nuclear counterstain 4′,6-diamidino-2-phenylindole (DAPI, 1:10 000) and the antifade reagent triethylenediamine (TDE, 2.5%). Due to the fragility of cryostat sections, washing steps prior to cover-slipping were omitted. Under these conditions, *FAST*-DiI fluorescence remained sharp and stable for up to one month at 4 °C. Tested alternatives included dry preparation (MM1), phosphate buffer (MM2; 0.1 M PB with 0.1% EDTA, 0.01% NaN₃), 50% glycerol in PB (MM3; 0.1 M PB with 0.1% EDTA, 0.01% NaN₃), and Mowiol^®^ 4-88 with glycerol replaced by m-xylylenediamine (MM4; MXDA; DiI-compatible chemical; Zhu et al. 2020). All of these alternative conditions resulted in rapid signal loss (Table 1).

### Alternative protocols

Standard and alternative approaches were compared as outlined in the Methods. Observations on labeling quality, tracer stability, tracing distance, and tissue or section integrity guided the selection of the standard protocol (Table 1).

**Table 1:** Comparison of the standard protocol with alternative approaches

Step	Standard protocol (SP)	Alternative Protocol	Observation
Fixation	Coronal sections, 4% FA, 2 h–6 d	AF1: Whole brain, 4% FA, 1 mth–2 yrs	AF1: Tracer uptake seemed impaired after prolonged fixation (B1–B2, *n* = 8), compared to SP short fixation (B3–B8, *n* = 28)
		AF2: Coronal sections, 3% PLP, 2–6 d	AF2: PLP (B7, *n* = 3) showed no advantage over FA (B7, *n* = 3)
			→ SP selected for reliable tracer uptake and simplicity
Electrophoresis	80 V, 106 h	EP1: 25–60 V, 24–48 h	EP1: Sparse labeling, tracing distance: 1.2–3 cm. (B1–B4; successful in *n* = 2/12)
		EP2: 100–200 V, ≤72 h	EP2: Sparse labeling, tracing distance: 2.5–3.3 cm. tissue damage >120 V (B5–B6; successful in *n* = 2/8)
		EP3: 80 V, 106–168 h	EP3: Numerous fibers, tracing distance: 1.2–6 cm. (B7–B8; successful in *n* = 9/12) → SP selected for optimal tracing distance
Embedding	–	AE1: Agarose	AE1: Limited tissue stability, tissue and section loss (B3–B6)
		AE2: Polyacrylamide	AE2: Slightly improved tissue stability and sectioning properties (B3–B6)
			→ SP required no embedding and avoided tissue and section loss
Sectioning	Cryostat, 20–30 μm	AS: Vibratome, 60–100 μm	AS: Smaller blocks → tissue loss; thicker sections → blurred signals → incomplete reconstruction (B3-B6)
			SP: Larger tissue blocks, thinner sections (B1–B2, B7–B8)
			→ SP selected for consecutive section and suitability for reconstruction
Storage	Cryostat sections, – 80 °C	ST1: Cryostat sections, –20 °C	ST1: Cryostat sections: Rapid *FAST*-DiI signal loss at –20 °C (B1, B2)
		ST2: Vibratome sections, in PB at 4 °C	ST2: Vibratome sections: *FAST*-DiI signal stable at 4 °C (B3–B6)
			SP: Cryostat sections: *FAST*-DiI signal stable long-term at – 80 °C (B2, B7–B8) → SP selected for consistent long-term stability
Mounting medium	Mowiol^®^ 4-88 with α-thioglycerol	MM1: Dry preparation	MM1–4: All resulted in rapid signal loss (B2, B7–B8).
		MM2: PB	SP: Preserved specific labeling for 1mth at 4 °C (B2, B7–B8).
		MM3: 50% glycerol in PB	→ SP selected for stable labeling
		MM4: Mowiol^®^ 4-88 with MXDA	

### Data analysis

**Fig. 3:**
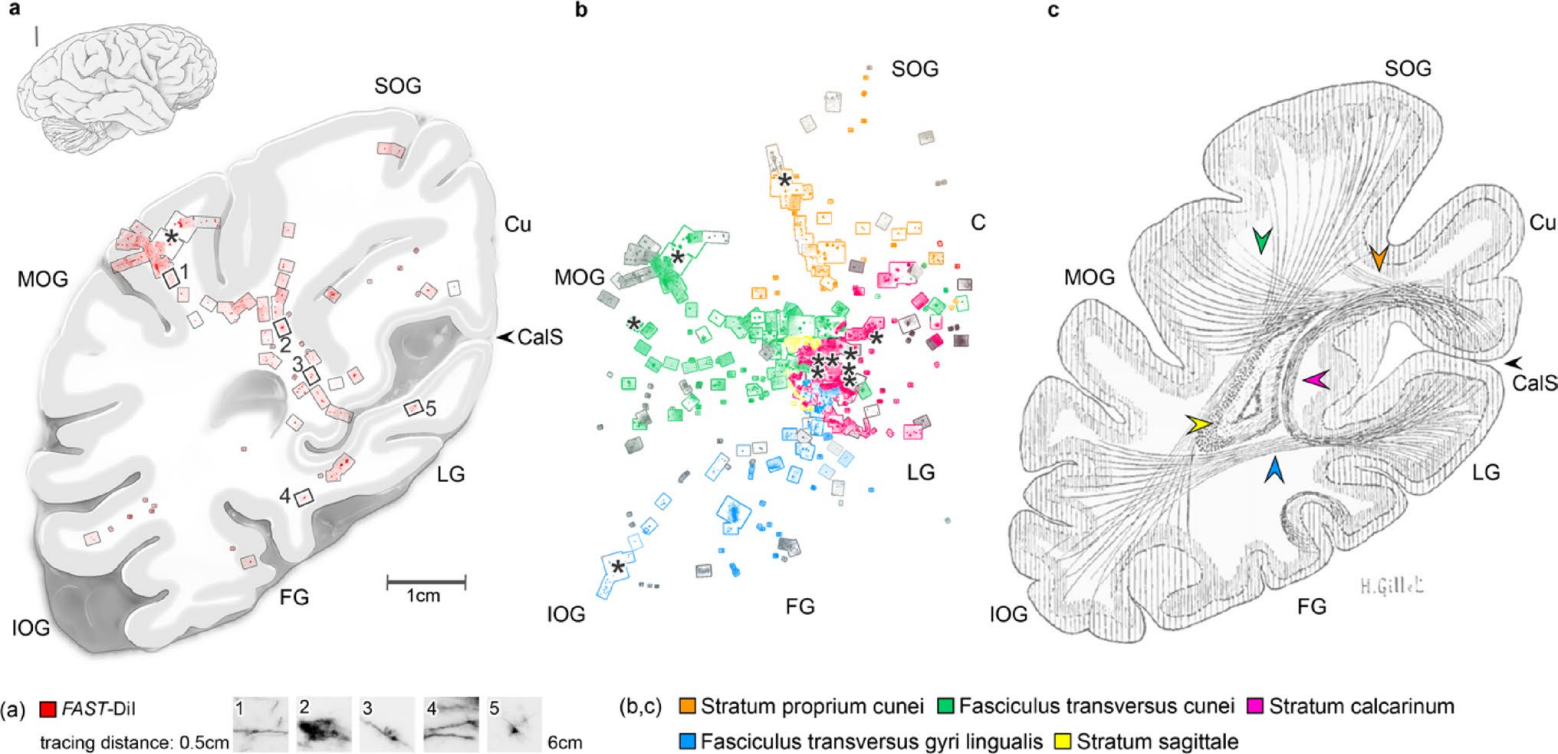
Overview of identified intralobar fibers in the human occipital lobe. **a** Tracer signal mapping from a single experiment following *FAST*-DiI application into the middle occipital gyrus (MOG), revealing labeled fibers extending to labeled cells in the calcarine cortex and cuneus. The most distant signal was detected 6 cm from the application site, measured along the shortest probable white matter pathway. Numbers 1–5 indicate the positions of microscopic images. **b** Summary of 11 experiments superimposed into one image, displaying only data points corresponding to intralobar fibers originating from or terminating in the calcarine cortex. **c** The findings are consistent with intralobar fiber tracts described by Déjerine and Déjerine-Klumpke (1895). Asterisks indicate tracer application sites

The sections were examined using a fluorescence microscope equipped with a digital camera (Hamamatsu ORCA-05G). The location of the detected signals was measured and transposed onto overview sketches using a millimeter grid. The course and orientation of labeled fibers in the white matter were analyzed to determine the shortest feasible and anatomically possible path between the tracer application site and the most distant signal. Cortical layers were identified by DAPI counterstaining. Hand-drawn sketches of transverse brain sections were created using photographs as references. Microscopic images were processed with ImageJ (inverted and contrast-enhanced), and figures were assembled using Adobe Photoshop. All figures represent the left hemisphere, with data from the right hemisphere mirrored accordingly. For signal mapping (Fig. 3), images were inverted, contrast was enhanced, dark areas were enlarged and contoured, false color-coding was applied, and the resulting images were projected onto the overview drawings.

## Results

To assess the performance of RFTE in human postmortem tissue, labeling specificity was first evaluated through control experiments to exclude unspecific diffusion or background signals. Tracing distances and reproducibility were analyzed to demonstrate that fibers could be followed over long trajectories. Finally, applications in the occipital lobe, including the calcarine cortex and adjacent gyri, provided validation against anatomical reference data and enabled the identification of distinct fiber pathways.

### Specificity of labeling

As expected, passive diffusion of *FAST*-DiI without electrophoresis did not result in significant transport beyond a few millimeters (Supplementary Fig. 1a). Following electrophoresis, no radial spread from the application site was observed (Supplementary Fig. 1b), labeling was consistently dependent on the tracer application site and confined to expected pathways. Incubation of occipital tissue in tracer-containing buffer resulted only in surface staining confined to layer I, without cellular or white-matter labeling (Supplementary Fig. 1c). Negative controls processed without tracer displayed only weak fluorescence background, attributable to erythrocytes, lipofuscin granules (Supplementary Fig. 1d), and the pia and arachnoidea mater (Supplementary Fig. 1e).

### Tracing distance and reproducibility

Across all successful electrophoretic tracing experiments (*n* = 13), labeled fibers were reconstructed over distances of 1.2–6 cm (mean: 3.4±0.8 cm), measured along the shortest anatomically plausible white-matter routes (Fig. 3a). Low-voltage, short runs (≤60 V, ≤48 h) produced only sparse labeling with tracing distances of 1.2–3 cm (successful in 2/12 experiments). High-voltage runs (100–200 V, ≤72 h) increased tracing distances to 2.5–3.3 cm (successful in 2/8 experiments) but were frequently associated with tissue damage. Under optimal conditions (80 V, 106–168 h, Table 1), labeling distances reached 1.2–6 cm and were reproducible across preparations (successful in 9/12 experiments), enabling continuous reconstruction across consecutive sections (Fig. 3a, b). Control experiments (80 V, 72 h) in the peripheral nervous system (PNS) yielded comparable results, with tracing distances of 3.7–4.9 cm (4.4±0.5 cm, *n* = 3; Supplementary Fig. 2). Compared to earlier approaches, RFTE achieves a five to six-fold increase in tracing distance and a 20-fold acceleration in diffusion speed (Supplementary Table 1).

### The four pathways of the calcarine cortex

Small deposits of *FAST*-DiI were placed in the calcarine cortex (CalCx) at varying depths within the calcarine sulcus (CalS): the upper bank (CalCx-Cu; *n* = 2), the floor (CalCx-F; *n* = 2, when present as a distinct deep bank), and the lower bank (CalCx-LG; *n* = 3). The resulting signal was detected in various regions, including the cuneus (Cu), and the inferior (IOG), middle (MOG), and superior (SOG) occipital gyri, as well as the lingual (LG) and fusiform (FG) gyri. Complementary tracer applications were performed at these identified projection sites within the IOG (*n* = 1), MOG (*n* = 2), and SOG (*n* = 1) to determine the cellular origins in the CalCx and the termination sites of reciprocal feedback projections. Stained fibers in the white matter were reconstructed from successive histological sections (Fig. 3a). Labeling was regarded as specific only when fibers could be reconstructed continuously across consecutive sections into a cortical target. In cases where cortical entry was not observed, labeling was accepted if fibers remained closely associated with the expected cortical region of origin or termination. Four fiber tracts originating from or terminating in the CalCx were identified (Fig. 3b; Table 2), consistent with historical descriptions (Fig. 3c): the *stratum proprium cunei* (SPC), the *fasciculus transversus cunei* (FTC), the *stratum calcarinum* (SC), and the f*asciculus transversus gyri lingualis* (FTL).

**Table 2:** Differential tract labeling after tracer applications in calcarine cortex and occipital gyri

Case	Application site	SPC	FTC	SC	FTL	VOF	SS
B3-L-S1	CalCx-Cu	+	±	±	-	-	-
B7-R-S2	CalCx-Cu	++	+++	++	-	-	(+)
B6-L-S2	CalCx-F	-	+	+	-	-	-
B7-L-S1	CalCx-F	-	+	-	-	-	-
B7-L-S2	CalCx-LG	-	-	++	-	-	(++)
B7-L-S3	CalCx-LG	-	-	++	+++	-	(+)
B5-L-S1	CalCx-LG	-	-	+	±	-	-
B8-R-S1	SOG	++	-	-	-	(±)	-
B8-R-S3	MOG	-	+++	-	-	-	-
B8-L-S2	MOG	-	(++)	-	-	-	-
B8-R-S1	IOG	-	-	-	++	±	-

Labeling scale along the entire pathway: – no labeling; ± sparse fibers; + few fibers; ++ moderate fibers; +++ numerous fibers; ( ) = no cortical entry observed

Abbreviations: SPC, *stratum proprium cune*; FTC, *fasciculus transversus cune*; SC, *stratum calcarinum*; FTL, *fasciculus transversus gyri lingualis*; VOF, *vertical occipital fascicle*; SS, *sagittal stratum*

Experiments are coded according to brain (B1–B8), hemisphere (L/R), and section number (S1–S3)

### The stratum proprium cunei

**Fig. 4:**
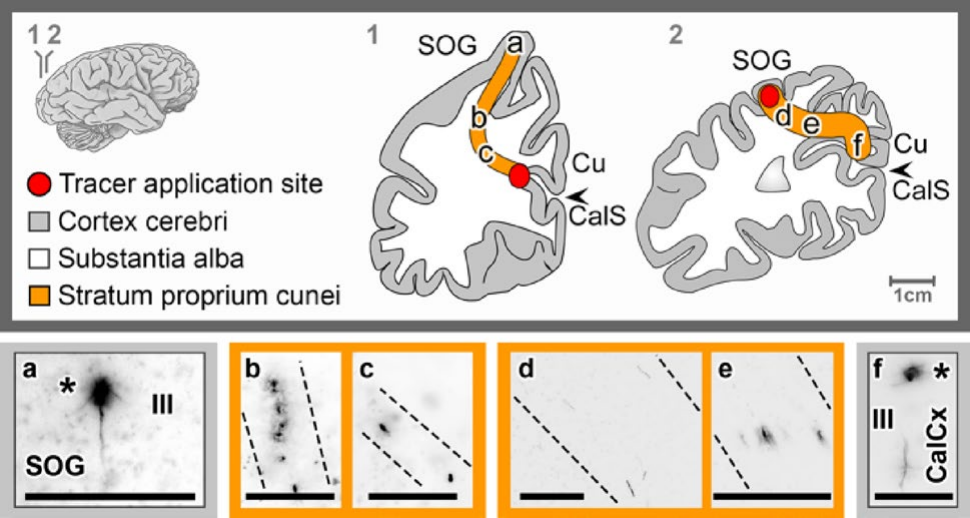
Tracing the stratum proprium cunei following *FAST*-DiI application to the cuneal (Cu) calcarine cortex (CalCx) (**a**–**c**) and superior occipital gyrus (SOG) (**d**–**f**), highlighting fiber pathways (dotted lines) and associated cell bodies (asterisk). Transverse brain sections at indicated levels. Scale bar: 100 μm

The proper stratum of the cuneus (*stratum proprium cunei*, SPC) is a short and narrow pathway, connecting the inferior aspect of the Cu with the superomedial aspect of the occipital lobe. Upon tracer application to the upper bank of the CalCx (*n* = 2), reciprocal connections were observed (Fig. 4a-c). In the SOG, radial ascending fibers reaching layers II and III and a retrogradely stained neuron in layer III were found (Fig. 4a). Upon complementary tracer application into the white matter of the SOG (*n* = 1), a thin fiber bundle was observed (Fig. 4d-e) that divided, in its course, into a direct, straighter tract projecting to layer III of the inferolateral CalCx, while other fibers bent toward the inferomedial CalCx, where a retrogradely labeled neuron in layer III could be found (Fig. 4f).

### The fasciculus transversus cunei

**Fig. 5:**
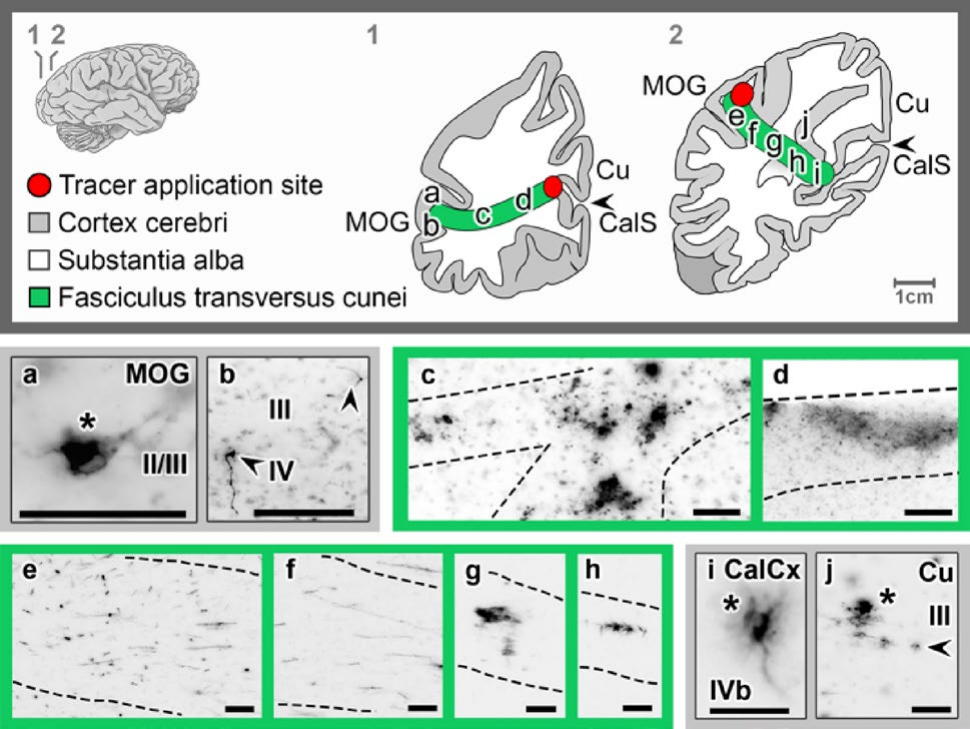
Tracing the fasciculus transversus cunei following *FAST*-DiI application to the cuneal (Cu) calcarine cortex (CalCx) (**a**–**d**) and middle occipital gyrus (MOG) (**e**–**j**), revealing fiber pathways (dotted lines), axons (arrowheads), and cell bodies (asterisks) in the MOG, CalCx and Cu. Transverse brain sections at indicated levels. Scale bar: 100 μm

The transverse fascicle of the cuneus (*fasciculus transversus cunei*, FTC) connects the inferior aspect of the Cu with the lateral surface of the occipital lobe. Upon tracer application into the upper bank (*n* = 2) and floor of the CalCx (*n* = 2), a distinct cord of fibers was observed which projected transversely and laterally to the MOG and IOG (Fig. 5a-d). In their course, they crossed U-shaped fibers which covered the floor of the CalS (Fig. 5a, f). Near the MOG, further ramifications were visible (Fig. 5c), which run in an inferior and lateral direction. In the MOG, fibers that terminated in layers III and IV as well as retrogradely labeled neurons in layer II/III were detected (Fig. 5a, b). Upon complementary tracer application into the white matter of the MOG (*n* = 2), the same fiber tract was traced (Fig. 5e-j) to labeled cells in layers III and IVb in the inferomedial CalCx and superolateral Cu, respectively (Fig. 5i, j).

### The stratum calcarinum

**Fig. 6:**
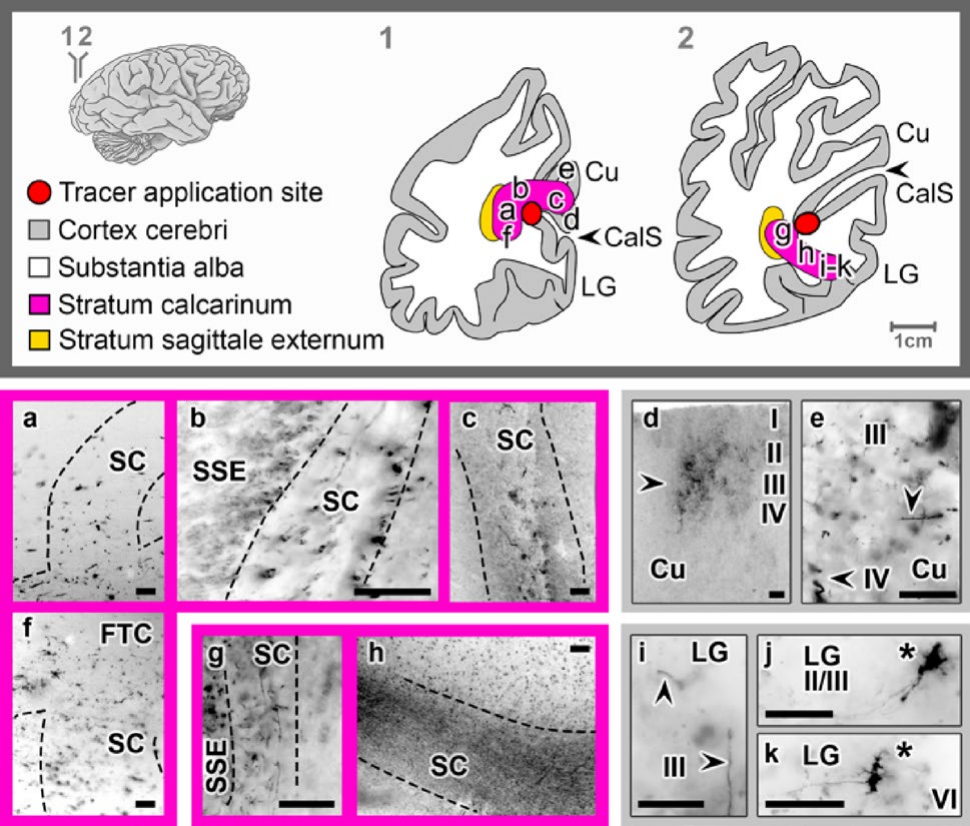
Tracing the stratum calcarinum following *FAST*-DiI application to the cuneal (Cu) (**a**–**f**) and lingual (LG) (**g**–**k**) regions of the calcarine cortex (CalCx), revealing labeled axons (arrowheads) in the Cu, as well as labeled axons and cell bodies (asterisks) in the LG. Transverse brain sections at indicated levels. Scale bar: 100 μm

The calcarine stratum (*stratum calcarinum*, SC) is a large bundle of fibers that interconnects the upper and lower edges of the CalCx. It consisted of a superficial layer with short projections that densely cover the floor of the CalS, giving it a U-shaped appearance, while the underlying layer had wider projections (Fig. 6a-k). Beneath the SC and adjacent to the posterior horn of the lateral ventricle, fibers running in a fronto-occipital direction could be observed (Fig. 6b, g). Based on their position and orientation, these fibers correspond to fibers of the external sagittal stratum (*stratum sagittale externum*, SSE). Tracer application into the upper bank of the CalCx (*n* = 2) showed superficial local projections to the inferomedial Cu and deeper projections to the superior aspects of the Cu. In the Cu, radial ascending fibers, as well as transversal fibers in layers II, III, and IV, could be observed (Fig. 6d, e). Additionally, tracer application to the lower bank of the CalCx (*n* = 3) revealed both superficial and deep projections to and from the posterior superolateral and inferolateral LG, respectively (Fig. 6g, h). In the inferolateral LG, radial ascending fibers in layers III and IV and retrogradely stained neurons in layers II/III and VI were found (Fig. 6i-k).

### The fasciculus transversus gyri lingualis

**Fig. 7:**
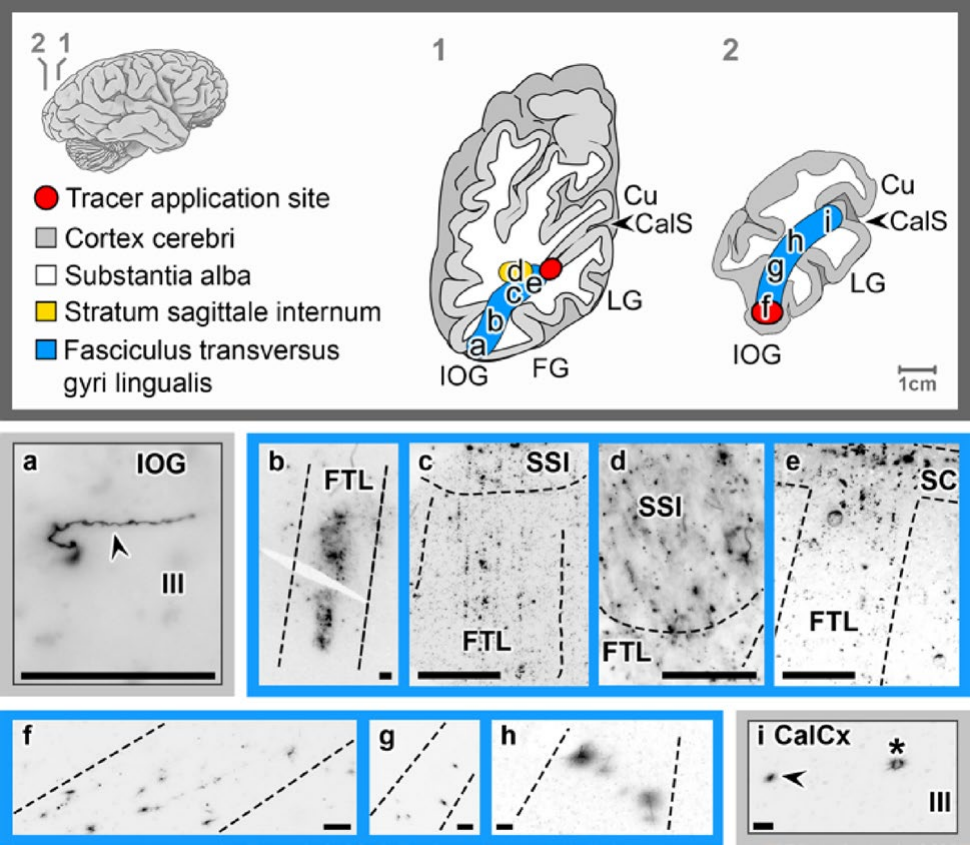
Labeling of the fasciculus transversus gyri lingualis following *FAST*-DiI application into the lingual (LG) (**a**–**e**) calcarine cortex (CalCx) and inferior occipital gyrus (IOG) (**f**–**i**), highlighting fiber pathways (dotted lines), axons (arrowheads), and cell bodies (asterisks) in the CalCx and IOG. Transverse brain sections at indicated levels. Scale bar: 100 μm

The transverse fascicle of the lingual gyrus (*fasciculus transversus gyri lingualis*, FTL) is hypothesized as fiber bundle that originates from the lower bank of the CalCx and the white matter of the LG. Upon tracer application into the lower bank of the CalCx (*n* = 2), fibers in the white matter were observed following an oblique course, some appearing to merge with other fiber tracts — i.e., the SC and the internal sagittal stratum (*stratum sagittale internum*, SSI) — before spreading out in layers IV to III in the FG and IOG (Fig. 7a-e). Complementary tracer application into the white matter of the IOG (*n* = 1) the same fiber tract was labeled and could be traced to cells in layer III of the inferolateral or lower bank of the CalCx (Fig. 7f-i).

## Discussion

Rotating Field Tracer Electrophoresis (RFTE) enabled the reconstruction of intercortical fibers in the postmortem human occipital lobe over distances not previously achieved with passive diffusion-based methods. Four association pathways linked to the calcarine cortex were delineated at cellular resolution. These observations are consistent with classical anatomical descriptions (Wernicke 1881; Sachs 1892; Vialet 1893a, b; Déjerine and Déjerine-Klumpke 1895) and with principles of hierarchical and reciprocal cortico-cortical organization derived from non-human primates (Felleman and Van Essen 1991). Taken together, the data supports RFTE as a practical approach for studying human intercortical connectivity.

### Association pathways of the calcarine cortex

**Fig. 8:**
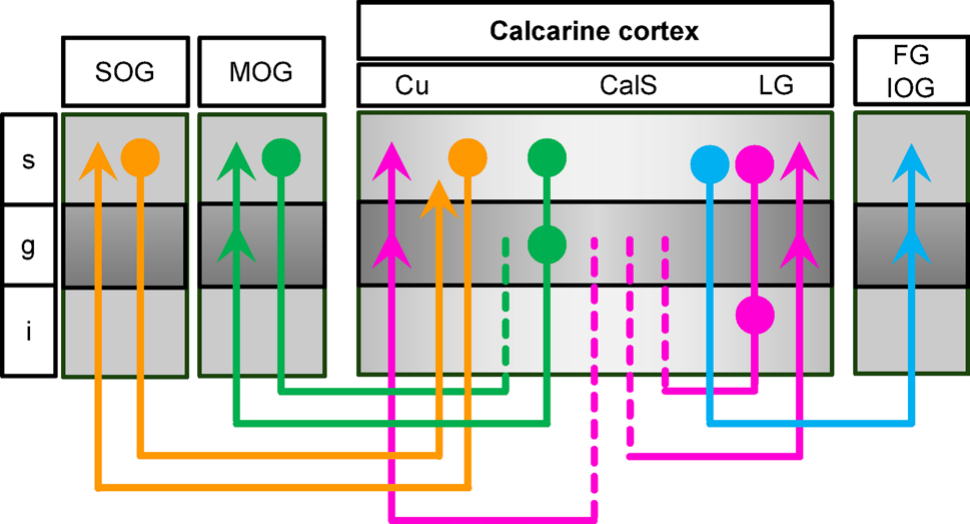
Schematic representation of connections between the calcarine cortex, and occipital, lingual and fusiform gyri originating (circles) and terminating (arrowheads) in the infragranular (i), granular (g), and supragranular (s) layers. Dotted lines indicate unresolved cellular origins or axonal termination

The *stratum proprium cunei* (SPC) has long been proposed as a short-range sagittal fiber system linking the upper calcarine cortex to the superior occipital gyrus (Sachs 1892; Vergani et al. 2014; Bugain et al. 2021; Forkel et al. 2015). While Bugain et al. (2021) noted similarities between this pathway, the *stratum calcarinum*, and the *sledge runner fascicle*, the present study provide evidence that allows the SPC to be distinguished from these adjacent systems by clarifying both its cortical origin and its termination sites (Figs. 4 and 8). It broadly fits the definition of feedforward and feedback projections.

The *fasciculus transversus cunei* (FTC) was previously identified as a horizontally oriented pathway linking the calcarine cortex with the middle and inferior occipital gyri. Although this tract was already demonstrated in a recent dissection study (Vergani et al. 2014), its cortical terminations remained unresolved. The present results support an origin in the calcarine cortex and cuneus, with terminations in the lateral occipital lobe (Figs. 5 and 8). This projection pattern is partly consistent with feedforward connectivity, but additional data will be required to establish its classification more firmly.

The *stratum calcarinum* (SC) consists of a large bundle of fibers interconnecting the upper and lower edges of the calcarine cortex (Sachs 1892; Bugain et al. 2021; Vergani et al. 2014). The observation of radial ascending fibers in the cuneus and lingual gyrus, together with retrogradely labeled cells of bilaminar origin, indicates reciprocal lateral connectivity (Figs. 6 and 8). It should be noted, however, that tracer applications were restricted to the calcarine cortex itself; as a result, fibers interlinking the centromedial aspects of the cuneus and lingual gyrus were not labeled.

The *fasciculus transversus gyri lingualis* (FTL) was observed as an intralobar occipito-occipital association pathway projecting to the fusiform and inferior occipital gyri. Its oblique trajectory and partial overlap with the *stratum calcarinum*, callosal, and projection fibers complicate its delineation with other methods. The present results, however, reveal supragranular projections terminating in the granular and supragranular layers of the fusiform and inferior occipital gyri, consistent with a feedforward projection (Figs. 7 and 8). While Schmahmann and Pandya (2009) suggested that these fibers may correspond to transverse fibers of Burdach’s (1822) inferior longitudinal fascicle (ILF), linking lateral and medial cortices in the occipital and temporal lobes, the evidence presented here supports the interpretation of the FTL as an independent occipito-occipital association tract.

### Methodological considerations

A central concern is whether RFTE produces pathway-specific labeling rather than indiscriminate dye spread. Here, several converging observations speak in favor of specificity and provide a falsifiable framework. (i) Differential outcomes: Tracer deposits placed in neighboring regions of the calcarine cortex (upper bank, floor, lower bank) were tract specific (Table 2). Upper bank applications labeled the *stratum proprium cunei* (SPC), the *fasciculus transversus cunei* (FTC), and the *stratum calcarinum* (SC), whereas lower bank applications labeled the *stratum calcarinum* (SC) and the *fasciculus transversus gyri lingualis* (FTL). Under a null hypothesis of non-specific spread, similar radial halos would be expected. (ii) Bidirectional reconstruction: Complementary applications in putative target gyri (SOG, MOG, IOG) labeled the same tracts and revealed retrogradely labeled neurons in the calcarine cortex. (iii) Laminar concordance: Termination patterns and retrogradely labeled cell bodies were confined to layers consistent with primate data. (iv) Internal negative control: In regions with major fiber crossings, labeling remained restricted to the expected trajectories. If RFTE had produced indiscriminate spread, frequent co-labeling of these prominent bundles would be expected. (v) Classical controls: Incubation in tracer-containing buffer yielded only surface staining restricted to layer I. Unstained tissue showed only expected autofluorescence. Together, these controls argue against artifactual background leading to tract-like patterns. On this methodological foundation, the observed results can be considered specific and valid.

### Conclusion

Overall, this study provides the first experimental evidence of intercortical association pathways in the human occipital lobe at the cellular level. Further research may clarify whether the distribution of projections and their cellular origins across cortical layers align with or differ from those observed in non-human primate studies (Felleman and Van Essen 1991). With its gain in tracing distance and speed, RFTE addresses a long-standing practical bottleneck in human postmortem tract tracing. The method complements dissection and diffusion-weighted imaging by resolving cellular origin and laminar terminations—features that are challenging to capture with non-invasive or macroscale approaches. In the future, adaptations such as multielectrode configurations for fully three-dimensional reconstruction may further extend the applicability of tracer electrophoresis and consolidate its role in mapping human cortical connectivity.

## Supplementary Information

Below is the link to the electronic supplementary material.

Supplementary file1

## Data Availability

No datasets were generated or analysed during the current study.

## References

[CR1] Bugain M, Dimech Y, Torzhenskaya N, de Schotten MT, Caspers S, Muscat R, Bajada CJ (2021) Occipital intralobar fasciculi: a description, through tractography, of three forgotten tracts. Commun Biology 4:433 10.1038/s42003-021-01935-3 PMC8010026 33785859

[CR2] Burdach KF (1822) Von Baue und leben des Gehirns. - Zweyter band. Leipzig, Dyk’sche Buchhandlung

[CR3] Burkhalter A, Bernardo K (1989) Organization of corticocortical connections in human visual cortex. Proc Natl Acad Sci USA 86:1071–1075 2464827 10.1073/pnas.86.3.1071 PMC286623

[CR4] Burkhalter A, Bernardo KL, Charles V (1993) Development of local circuits in human visual cortex. J Neurosci 13:1916–1931 8478684 10.1523/JNEUROSCI.13-05-01916.1993 PMC6576577

[CR5] Caspers S, Axer M, Caspers J, Jockwitz C, Jütten K, Reckfort J, Grässel D, Katrin Amunts, and, Zilles K (2015) Target sites for transcallosal fibers in human visual cortex – A combined diffusion and polarized light imaging study. Cortex 72:40–53 25697048 10.1016/j.cortex.2015.01.009

[CR6] Chen BK, Miller SM, Mantilla CB, Gross L, Yaszemski MJ, Windebank AJ (2006) Optimizing conditions and avoiding pitfalls for prolonged axonal tracing with carbocyanine dyes in fixed rat spinal cords. J Neurosci Methods 154:256–263 16466800 10.1016/j.jneumeth.2005.12.025

[CR7] Déjerine J, Déjerine-Klumpke A (1895) Anatomie des centres nerveux (Paris, Rueff et cie)

[CR8] Felleman DJ, Van Essen DC (1991) Distributed hierarchical processing in the primate cerebral cortex. Cereb Cortex 1:1–47 1822724 10.1093/cercor/1.1.1-a

[CR9] Forkel SJ, Mahmood S, Vergani F, Catani M (2015) The white matter of the human cerebrum: part I the occipital lobe by Heinrich Sachs. Cortex 62:182–202 25527430 10.1016/j.cortex.2014.10.023 PMC4298656

[CR11] Friedman DI, Johnson JK, Chorsky RL, Stopa EG (1991) Labeling of human retinohypothalamic tract with the carbocyanine dye, dii. Brain Res 560:297–302 1760734 10.1016/0006-8993(91)91246-w

[CR12] Godement P, Vanselow J, Thanos S, Bonhoeffer F (1987) A study in developing visual systems with a new method of staining neurones and their processes in fixed tissue. Development 101:697–713 2460302 10.1242/dev.101.4.697

[CR13] Hayaran A, Bijlani V (1992) Polyacrylamide as an infiltrating and embedding medium for vibratome sectioning of human fetal cerebellum containing Dil-filled axons. J Neurosci Methods 42:65–68 1405734 10.1016/0165-0270(92)90135-z

[CR14] Hildebrand S, Schueth A, von Wangenheim K, Mattheyer C, Pampaloni F, Bratzke H, Roebroeck AF, Galuske RAW (2020) hFRUIT: an optimized agent for optical clearing of DiI-stained adult human brain tissue. Sci Rep 10:9950 32561795 10.1038/s41598-020-66999-3 PMC7305111

[CR15] Hilgetag CC, Medalla M, Beul SF, Barbas H (2016) The primate connectome in context: principles of connections of the cortical visual system. NeuroImage 134:685–702 27083526 10.1016/j.neuroimage.2016.04.017 PMC5135480

[CR16] Hofmann MH, Bleckmann H (1999) Effect of temperature and calcium on transneuronal diffusion of dii in fixed brain preparations. J Neurosci Methods 88:27–31 10379576 10.1016/s0165-0270(99)00007-2

[CR17] Holmqvist BI, Ostholm T, Ekström P (1992) DiI tracing in combination with immunocytochemistry for analysis of connectivities and chemoarchitectonics of specific neural systems in a teleost, the Atlantic salmon. J Neurosci Methods 42:45–63 1383644 10.1016/0165-0270(92)90134-y

[CR18] Isaacson MD, Hedwig B (2017) Electrophoresis of Polar fluorescent tracers through the nerve sheath labels neuronal populations for anatomical and functional imaging. Sci Rep 7:40433 28084413 10.1038/srep40433 PMC5233955

[CR19] Latini F, Hjortberg M, Aldskogius H, Ryttlefors M (2015) ‘The Classical Pathways of Occipital Lobe Epileptic Propagation Revised in the Light of White Matter Dissection’, *Behavioural Neurology*, 2015: 872645 10.1155/2015/872645 PMC4430656 26063964

[CR20] Lukas JR, Aigner M, Denk M, Heinzl H, Burian M, Mayr R (1998) Carbocyanine postmortem neuronal tracing. Influence of different parameters on tracing distance and combination with immunocytochemistry. J Histochem Cytochem 46:901–910 9671441 10.1177/002215549804600805

[CR21] Madison RD, Robinson GA, Krarup C, Moldovan M, Li Q, Wilson WA (2014) In vitro electrophoresis and in vivo electrophysiology of peripheral nerve using DC field stimulation. J Neurosci Methods 225:90–96 24485870 10.1016/j.jneumeth.2014.01.018 PMC3971989

[CR22] Makarenko IG, Ugrumov MV, Calas A (2001) Axonal projections from the hypothalamus to the median eminence in rats during ontogenesis: dii tracing study. Anat Embryol 204:239–252 10.1007/s004290100181 11681803

[CR23] McCasland JS, Woolsey TA (1988) New high-resolution 2-deoxyglucose method featuring double labeling and automated data collection. J Comp Neurol 278:543–554 3068265 10.1002/cne.902780406

[CR24] McLean IW, Nakane PK (1974) Periodate-lysine-paraformaldehyde fixative a new fixative for immunoelectron microscopy. J Histochem Cytochemistry 22:1077–1083 10.1177/22.12.1077 4374474

[CR25] Mufson EJ, Brady DR, Kordower JH (1990) Tracing neuronal connections in postmortem human hippocampal complex with the carbocyanine dye dii. Neurobiol Aging 11:649–653 1704107 10.1016/0197-4580(90)90031-t

[CR26] Murphy MC, Fox EA (2007) Anterograde tracing method using dii to label vagal innervation of the embryonic and early postnatal mouse Gastrointestinal tract. J Neurosci Methods 163:213–225 17418900 10.1016/j.jneumeth.2007.03.001 PMC1974840

[CR27] Rushmore RJ, Bouix S, Kubicki M, Rathi Y, Yeterian EH, Makris N (2020) How human is human connectional neuroanatomy? Front Neuroanat 14:18 32351367 10.3389/fnana.2020.00018 PMC7176274

[CR28] Sachs H (1892) Das Hemisphärenmark des menschlichen Grosshirns. Leipzig, Georg Thieme

[CR29] Schmahmann JD, Pandya D (2009) Fiber pathways of the brain. OUP USA

[CR30] Sparks DL, Lue LF, Martin TA, Rogers J (2000) Neural tract tracing using Di-I: a review and a new method to make fast Di-I faster in human brain. J Neurosci Methods 103:3–10 11074091 10.1016/s0165-0270(00)00291-0

[CR31] Swift MJ, Crago PE, Grill WM (2005) Applied electric fields accelerate the diffusion rate and increase the diffusion distance of dii in fixed tissue. J Neurosci Methods 141:155–163 15585299 10.1016/j.jneumeth.2004.06.011

[CR32] Takemura H, Pestilli F, Weiner KS (2019) Comparative neuroanatomy: integrating classic and modern methods to understand association fibers connecting dorsal and ventral visual cortex. Neurosci Res 146:1–12 30389574 10.1016/j.neures.2018.10.011 PMC6491271

[CR33] Tardif E, Clarke S (2001) Intrinsic connectivity of human auditory areas: a tracing study with dii. Eur J Neurosci 13:1045–1050 11264678 10.1046/j.0953-816x.2001.01456.x

[CR34] Thal DR, Capetillo-Zarate E, Galuske RA (2008) Tracing of temporo-entorhinal connections in the human brain: cognitively impaired argyrophilic grain disease cases show dendritic alterations but no axonal Disconnection of temporo-entorhinal association neurons. Acta Neuropathol 115:175–183 18075746 10.1007/s00401-007-0330-6 PMC2668579

[CR35] Vergani F, Mahmood S, Morris CM, Mitchell P, Forkel SJ (2014) Intralobar fibres of the occipital lobe: A post mortem dissection study. Cortex 56:145–156 24768339 10.1016/j.cortex.2014.03.002

[CR36] Vialet N (1893a) Les centres cérébraux de La vision et La̓ppareil nerveux visuel intra-cérébral. Félix Alcan, Paris

[CR37] Vialet N (1893b) Sur l’existence à la partie inférieure du lobe occipital un faisceau association distinct, le faisceau transverse du lobule lingual. Comptes rendus hebdomadaires des séances et mémoires de la Société de biologie: 793-95

[CR38] Wernicke C (1881) Lehrbuch der Gehirnkrankheiten für Aerzte und Studirende. v.1, 1881 (Fischer)

[CR39] Zhu J, Yu T, Li Y, Xu J, Qi Y, Yao Y, Ma Y, Wan P, Chen Z, Li X, Gong H, Luo Q, Zhu D (2020) MACS: rapid aqueous clearing system for 3D mapping of intact organs. Adv Sci 7:1903185 10.1002/advs.201903185 PMC7175264 32328422

