# Supplementary material for: Mapping intralobar fiber connections in the human occipital lobe by tracer electrophoresis
Source: Brain Struct Funct. 2026 Jan 6;231(1):8. doi: 10.1007/s00429-025-03031-2 (PMC12774988; doi:10.1007/s00429-025-03031-2)
Supplement: Supplementary file 1 — Supplementary file1 [file 429_2025_3031_MOESM1_ESM.docx]

***Supplementary information***

Supplementary Fig. 1. Control experiments.

Supplementary Fig. 2. *FAST*-DiI tracing over a 4.7cm distance within 72 hours in human peripheral nerve.

Supplementary Tab. 1. Comparative Analysis of Key Variables in the Staining Protocol with Lipophilic Dyes

**Supplementary Table 1. Comparative Analysis of Key Variables in the Staining Protocol with Lipophilic Dyes**

| **Paper** | **species** | **tissue** | **post-mortem delay**^+^ | **fixation** | **tracer** | **incubation** | **sectioning method** | **mounting** | **tracing distance** | **our inferences** |
| --- | --- | --- | --- | --- | --- | --- | --- | --- | --- | --- |
| Tardif and Clarke (2001) | human | CNS:  cortex | 4-23h | 4% PFA,  2h | DiI,  in DMF | 6-12mth | agarose, vibratome | 4% PFA | 5mm | - excellent DiI signal with PFA and short fixation |
| Mufson, Brady, and Kordower (1990) | human | CNS:  cortex | 4.5h | 4% PFA,  24h | DiI,  crystal | 6mth,  RT | -  vibratome | Apathy's mounting medium | 8mm | - excellent DiI signal with short postmortem delay and PFA |
| Hofmann and Bleckmann (1999) | goldfish | CNS | 0 | 4% PFA,  0,1% ETDA,  1-2d | DiI,  crystal | 1-8wk,  6-40°C | none, gelatin, vibratome | - | - | - calcium and heat facilitate fading and smearing of DiI labeling - EDTA prevents fading and smearing - glutaraldehyde does not improve labeling |
| Sparks et al. (2000) | pig, human | CNS:  brainstem, cerebellum | 2h / - | delayed fixation,  4% PFA,  5d | *FAST*-DiI,  in DMF | 36h prior to fixation, PBS,  4°C | -  vibratome, cryostat | - | 20-40mm | - max. *FAST*-DiI tracing distance of 40mm with delayed fixation - good signal in Yorkshire pig - diffuse smearing of DiI labeling after cryosectioning |
| Makarenko, Ugrumov, and Calas (2001) | rat,  embryo | CNS:  diencephalon | - | 4% PFA,  RT,  over night | DiI,  crystal | 2wks-2yrs, 4% PFA | none  vibratome,  sucrose,  cryostat | PBS,  dried | - | - vibratome sections can be stored in PFA at 4°C for 2 years without DiI signal deterioration - DiI signal deteriorates within 3 days in dried cryosections |
| Lukas et al. (1998) | guinea pig, human | PNS,  CNS:  spinal cord | 24-72h | 2-4% PFA | DiI,  DiO,  DiA,  crystal | 3wk-1+yr, 4% PFA,  37-40°C | sucrose,  cryostat,  -  vibratome | none, dried | ­~29.2mm  in guinea pigs  ~28.5mm  in human | - among DiI, DiA and DiO, DiI seems to be the best distance tracer - DiI tracing distance of max. ~31mm after 15wks at 37°C in PNS - temperatures higher than 37°C do not result in longer tracing distances - no differences in tracing distance between adult and newborn animals - slight difference in tracing distance between animal and human tissue - fading after drying of cryosections |
| Hildebrand et al. (2020) | porcine, human | CNS:  cortex | 12h | 2.6% PLP,  0.8% iodoacetic acid,  2-7d | DiI,  crystal | 6mth-1+yr, 2% PFA,  37°C | whole mount | hfRUit | 0.4mm | - hfRUit optical clearing compatible with DiI - PLP fixative is considered to facilitate the diffusion of DiI - DiI signals were well maintained with α-Thioglycerol |
| Godement et al. (1987) | mouse: embryo,  neonatal  chicken: embryo  rat: adult | CNS:  optic nerve,  retina | 0 | 4-10% PFA,  1-2% glutaraldehyde,  1-3d | DiI,  DiO,  crystal | 1d-3mth, PFA,  RT / 4°C,  live animal: 48h,  then fixation | -  vibratome | - | - | - glutaraldehyde increases background and does not improve signal - cryosectioning should be avoided - transcellular labeling of cells in embryos - 10% PFA fixation limits diffusion |
| Burkhalter, Bernardo, and Charles (1993) | human | CNS:  cortex | - | 2.6% PLP,  0.8% iodoacetic acid,  1-2d | DiI,  crystal | 1-6mth, PB, 21°C | -  vibratome | PB | ~ 5mm* | - good DiI signal with PLP - vibratome sections can be stored in PB after analysis |
| Burkhalter and Bernardo (1989) | human | CNS:  cortex | 14-30h | 3% PLP,  0.8% iodoacetic acid, 4°C,  24h | DiI,  crystal | 2-8wk, PB, 21°C | -  vibratome | Aquamount Polysciences | ~ 7mm* | - good DiI signal with PLP, reason for PLP not specified, revers to McCasland and Woolsy in press (same institute) |
| Thal, Capetillo-Zarate, and Galuske (2008) | human | CNS:  cortex | 6-23h | 2.6% PLP,  0.8% iodoacetic acid, 5d | DiI,  crystal | 12mth,  PFA,  37°C | -  vibratome | TBS | 6-9.5mm | - max. tracing distance of 9.5mm observed in human cortex |
| Swift, Crago, and Grill (2005) | human | PNS | - | 10% PFA | DiI,  DiO, DiA,  DiR,  in EtOH | applied electric field,  72h,  mineral oil | polyacrylamide,  vibratome | - | 53.7mm | - electrophoresis increases DiI tracing distance and speed - no transaxonal diffusion |
| Friedman et al. (1991) | human | CNS:  optic nerve | 24h | 4-10% PFA,  37°C,  3-4mth | DiI  crystal | 3-4mth,  4% PFA,  37°C | -  vibratome | Gel-mount, Biomeda | - | - fading of signal in vibratome sections within 72h - storing of vibratome sections at 4°C extends shelf life - 10% Formalin-fixed brains may be less suitable |
| Holmqvist, Ostholm, and Ekström (1992) | salmon | CNS:  optic nerve  diencephalon,  metencephalon | 0h | 4% PFA,  0.1% glutaraldehyde | DiI  crystal | 1-10wks,  4% PFA, 40°C. | agarose,  vibratome  sucrose,  cryostat | PB,  PBS | - | - slightly better preservation of DiI labeling by postfixation with 0.1% glutaraldehyde - cryosectioning of DiI labeled tissue results in loss of staining and spread of dye - Dil is transported relatively rapidly in fixed tissue (1-10 weeks) |
| Hayaran and Bijlani (1992) | human: embryo,  fetus | CNS:  brainstem, cerebellum | - | 4% PFA,  4-6 wks | DiI  crystal | 2-16wks, 4% PFA,  RT | polyacrylamide,  vibratome | H_2_0 | - | - polyacrylamide as an infiltrating and embedding medium for vibratome sectioning of fragile tissue |
| Murphy and Fox (2007) | mice:  embryo,  neonatal | PNS:  vagal nerve | 0 | 4% PFA  0,1% ETDA,  4°C,  3d | DiI, crystal,  dried oil | 1–7wks, PBS,  37°C | whole-mount | PBS,  glycerol | - | - long incubation periods lead to DiI leakage - calcium facilitates DiI diffusion out of the membrane and promotes unspecific labeling - EDTA in the fixative reduces leakage - glycerol promotes leakage and loss of specific labeling |
| Chen et al. (2006) | rat | CNS:  spinal cord | 0 | 1-4% PFA,  24h | DiI,  DiO,  coated pins | 2-24wks,  1-4% PFA,  RT / 37°C | agarose,  vibratome | Gel-mount, Biomeda | 8.89  ±0.23mm | - stronger and longer-distance DiI and DiO staining at 37°C than at RT - DiI diffused significantly longer distances than DiO - no significant difference between 1% and 4% PFA fixation |
| Zhu et al. (2020) | mouse | organs:  vascular system |  | 4% PFA  4°C  over night | DiI,  CM-DiI  in EtOH | MACS  with MXDA | whole-mount, vibratome | MACS  with MXDA | - | - DiI and CM-DiI signals were well maintained with MXDA |
| McLean and Nakane (1974) | mouse | kidney, intestine,  liver | - | 0-4% PLP | - | - | sucrose,  cryostat | - | - | - PLP stabilizes carbohydrate moieties, carbohydrates are oxidized by periodate and cross-linked by lysine - PLP developed for immunoelectron microscopy |
| McCasland and Woolsey (1988) | mice | CNS | 0 | PLP  0.8% iodoacetic acid, 0°C,  over night | - | - | sucrose,  cryostat | - | - | - glycolytic inhibitor iodoacetic acid prevents glycogenolysis |

^+^ following determination of death; - not specified / does not apply; * approximated by scalebar


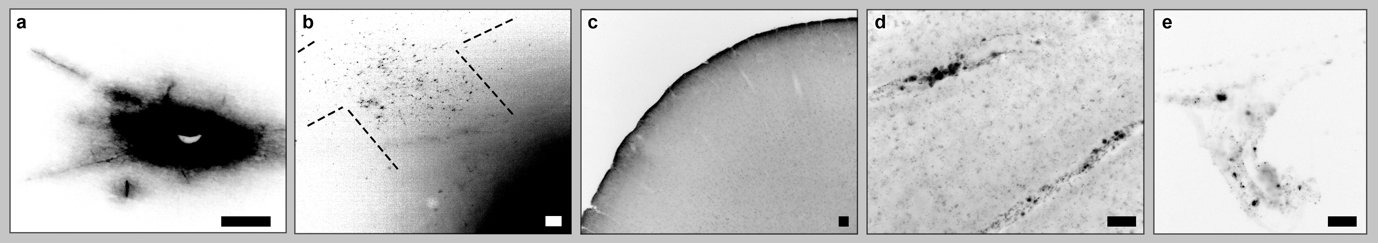


**Supplementary Figure 1. Control experiments.** (a) Passive diffusion of *FAST*-DiI without electrophoresis. (b) *FAST*-DiI electrophoresis shows no radial spread from the appllication site site. (c) Incubation in tracer-containing buffer only shows surface staining restricted to layer I. (d) Autofluorescence of erythrocytes and lipofuscin granules. (e) Autofluorescence of pia and arachnoid mater. Scale bars: 100μm.


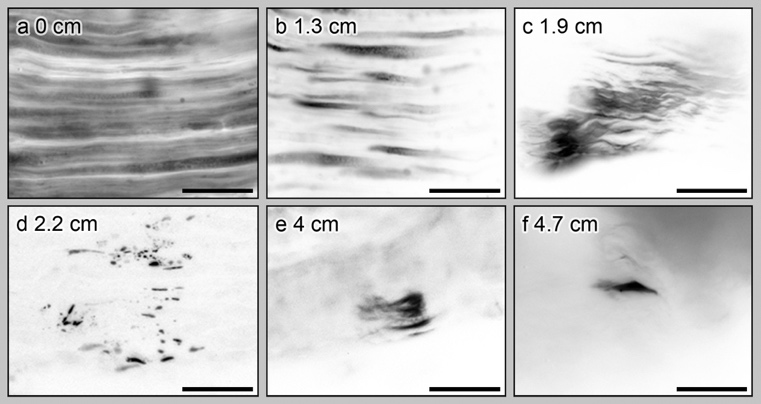


**Supplementary** **Fig. 2: *FAST*-DiI tracing over a 4.7cm distance within 72h at 80V in the human peripheral nerve.** Microscopic images (a-f) of longitudinal sections of the tibial nerve following *FAST*-DiI application and tracer electrophoresis. Stained axons and myelin sheaths are visible at the application site (a, 0 cm), with a progressively reduced number of labeled axons along the nerve (b-f, 1.3 - 4.7 cm). Scale bars: 100μm.
